# Building a Solid Foundation: The Need for Causal Evidence Before Advancing Anti‐Obesity Drug Development Targeting the Endocannabinoid System

**DOI:** 10.1002/prp2.70056

**Published:** 2024-12-24

**Authors:** Andrej Belančić, Farideh A. Javid

**Affiliations:** ^1^ Department of Basic and Clinical Pharmacology with Toxicology, Faculty of Medicine University of Rijeka Rijeka Croatia; ^2^ Department of Pharmacy, School of Applied Sciences University of Huddersfield Huddersfield UK

**Keywords:** anti‐obesity drugs, drug development, endocannabinoid system, obesity

We would like to commend the authors, Kurtov et al., for their insightful contribution published in *Pharmacology Research and Perspectives* on the potential of targeting the endocannabinoid system (ECS) in the regulation of appetite and the treatment of obesity [1]. Their thorough exploration of the role of ECS in obesity highlights the growing interest in this complex neuromodulatory system as a potential therapeutic target.

The purpose of this letter is to draw a parallel attention between their article and the recent review by Nele Mattelaer et al., published in the *International Journal of Obesity* [2]. Both contributions provide a valuable perspective, yet there is a critical gap in understanding that requires attention before advancing to novel drug therapies.

Mattelaer et al. conducted a comprehensive review of 56 human studies examining changes in circulating endocannabinoid levels after weight loss, either surgically, or through lifestyle modifications. They noted conflicting results, particularly with respect to 2‐arachidonoylglycerol (2‐AG) and arachidonoylethanolamine (AEA). Although AEA was positively correlated with body mass index (BMI), most studies did not find an association between 2‐AG and BMI. Importantly, few studies have explored the effects of pharmacological treatments on ECS modulation, and only one study showed no significant change in AEA levels after weight management interventions. In addition, studies on bariatric surgery yielded mixed results regarding ECS regulation.

Before we can confidently develop new drugs targeting the ECS, a causal relationship between ECS modulation and obesity must be established through rigorous randomized controlled trials (RCTs). This is particularly important given the emergence of new pharmacological agents such as tirzepatide, which has shown promising results in weight loss and could provide key information on the role of ECS. The variability in the findings in the current literature underscores the need for more focused RCTs that investigate how both pharmacological (with drugs such as tirzepatide) and surgical interventions influence the ECS. What is more, given the strong interconnection between obesity and psychological complications, potential confounding factors, such as underlying mental health issues, must be carefully addressed in ECS study designs.

Kurtov et al. rightly suggest that targeting the ECS may represent a unique therapeutic approach for the treatment of obesity. However, as Mattelaer et al. point out, it is essential that we first identify a clear causal link between ECS activity and obesity through well‐designed clinical trials. Once this causality is confirmed, we can then confidently advance toward developing therapies that modulate the ECS as a means of addressing the obesity pandemic.

In conclusion, the time is ripe for RCTs exploring the relationship between ECS modulation and weight loss, particularly in the context of new pharmacological agents. Only with this evidence can we truly assess the therapeutic potential of targeting ECS and determine whether to proceed with clinical trials, as success rates could be significantly affected without a solid foundation [3]. Currently, we await the forthcoming results on the effectiveness and safety of a novel cannabinoid receptor‐1 (CB1R) inverse agonist, INV‐202 [4, 5].

## Author Contributions

A.B. and F.A.J. contributed equally on all aspects, including conceptualization, investigation, writing, review, and editing. Both authors have read and agreed to the published version of the manuscript.

## Conflicts of Interest

The authors declare no conflicts of interest.

## Data Availability

The authors have nothing to report.
